# Tart Cherry Prevents Bone Loss through Inhibition of RANKL in TNF-Overexpressing Mice

**DOI:** 10.3390/nu11010063

**Published:** 2018-12-29

**Authors:** Nicholas Moon, Linda Effiong, Lee Song, Thomas R. Gardner, Do Y. Soung

**Affiliations:** 1 Department of Medicine, Case Western Reserve University, Cleveland, OH 44104, USA; Yxm273@case.edu; 2 Department of Orthopaedic Surgery, Columbia University, New York, NY 10032, USA; lae2133@cumc.columbia.edu (L.E.); sl705@cumc.columbia.edu (L.S.); trg1@cumc.columbia.edu (T.R.G.); 3 The Institutes of Food, CJ CheilJedang, CJ Blossom Park, 42, Gwanggyo-ro, Yeondong-gu, Suwon-si, Gyeonggi-do 16495, Korea

**Keywords:** tart cherry, rheumatoid arthritis, TNF, osteoblast, osteoclast, RANKL, OPG, Runx2, bone mass, infliximab

## Abstract

Current drugs for the treatment of rheumatoid arthritis-associated bone loss come with concerns about their continued use. Thus, it is necessary to identify natural products with similar effects, but with fewer or no side effects. We determined whether tart cherry (TC) could be used as a supplement to prevent inflammation-mediated bone loss in tumor necrosis factor (*TNF*)-overexpressing transgenic (TG) mice. TG mice were assigned to a 0%, 5%, or 10% TC diet, with a group receiving infliximab as a positive control. Age-matched wild-type (WT) littermates fed a 0% TC diet were used as a normal control. Mice were monitored by measurement of body weight. Bone health was evaluated via serum biomarkers, microcomputed tomography (µCT), molecular assessments, and mechanical testing. TC prevented TNF-mediated weight loss, while it did not suppress elevated levels of interleukin (IL)-1β and IL-6. TC also protected bone structure from inflammation-induced bone loss with a reduced ratio of receptor activator of nuclear factor kappa-B ligand (RANKL)/osteoprotegerin (OPG) to a degree comparable to infliximab. Furthermore, unlike with infliximab, TC exhibited a moderate improvement in TNF-mediated decline in bone stiffness. Thus, TC could be used as a prophylactic regimen against future fragility fractures in the context of highly chronic inflammation.

## 1. Introduction

Rheumatoid arthritis (RA) is a chronic inflammatory disease resulting in joint destruction [1]. RA patients also exhibit low bone mass and increased risk of fracture compared to a representative population [2,3,4,5,6,7]. Bone loss and fragility in RA patients have been attributed to inactivity and use of corticosteroids for treating RA. However, inflammation has also been suggested as a risk factor [2,8,9,10,11,12,13]. 

Inflammatory cytokines, particularly tumor necrosis factor (TNF), are considered a contributing mediator in RA-associated bone loss. Specifically, TNF increases expression of the receptor activator of nuclear factor κB ligand (RANKL), which binds to RANK on the surface of pre-osteoclasts to generate mature osteoclasts, the bone resorbing cells [14]. On the other hand, TNF inhibits building of the bone matrix by decreasing differentiation of osteoblasts, along with downregulating gene expression of alkaline phosphatase (AP) and type I collagen (COL I) [14]. In fact, RA-associated bone loss is effectively treated by TNF-targeting antibodies such as infliximab, etanercept, or adalimumab [2,8,9,10,11,15,16]. Unfortunately, a significant side effect of these drugs is immunosuppression that leaves the patient greatly susceptible to infection [17]. Thus, developing alternative TNF-targeting strategies using an inflammation-modifying food that does not render the patient severely immunocompromised would be ideal for RA patients with osteoporosis. 

One such candidate is tart cherry (TC, *Prunus cerasus*), a fruit rich in flavonoids such as flavonols (i.e., quercetin), hydroxycinnamic acids (i.e., neochlorogenic acid), flavan-3-ols (i.e., proanthocyanidins), and anthocyanidins [18,19]. TC with high content of these polyphenols has been reported to display antioxidant and anti-inflammatory properties [19,20,21,22,23,24,25,26,27,28]. For example, TC is capable of improving osteoarthritis by reducing C-reactive protein, a serum marker of inflammatory state, in a clinical trial [29]. Emerging data have also revealed that symptoms of RA are attenuated by treatments with individual polyphenol ingredients of TC including quercetin, proanthocyanidins, and antocyanidins in mouse models of RA using either collagen or adjuvant induction [30,31,32]. 

For these reasons, TC could be a promising non-pharmaceutical substitute for the drugs used in the prevention of bone loss in the setting of inflammation. Through this study, we evaluated the dose-dependent effects of TC diet on inflammation-mediated bone loss using TNF-overexpressing transgenic (TG) mice. 

## 2. Materials and Methods

### 2.1. Animals and Diet

Female 3.5–4.5 week-old TG mice [33] were randomly assigned to three groups to feed on a diet of 0%, 5%, or 10% TC for 4 weeks. TG mice have been extensively characterized to be proper animal models of rheumatoid arthritis-associated bone loss [34,35] as well as joint destruction [14,36,37]. As a control for standard TNF treatment [13], TG mice were fed a 0% TC diet and treated with an intraperitoneal dose of 10 mg/kg infliximab twice per week. Age-matched wild-type (WT) mice fed on a diet of 0% TC diet were used as an intact control group. All diets were formulated to be comparable in macronutrient and calorie content (Table 1) based on an AIN-93G diet for growth (Harlan, Indianapolis, IN, USA). These diets also took into account the nutrition facts of TC that were provided by the Cherry Market Institute. Diets were given to mice by pair-feeding to equal amounts of consumption as previously described [14]. Mice were allowed to freely access water during the entire experiment. All mice were housed at the Columbia University Medical Center animal facilities according to state and federal animal care guidelines (Institutional Animal Care and Use Committee number: AC-AAAI1502). 

### 2.2. Inflammatory Cytokine Assay

After a four-week treatment, mice were fasted for at least 6 h prior to euthanasia. Mice were then anesthetized using 100 μl of intraperitoneal injection with 100 μg/kg of ketamine and 10 μg/kg of xylazine to obtain whole blood through cardiac puncture. Serum separated from blood was used to measure human TNF (hTNF), mouse interleukin-1β (mIL-1β), and mIL-6 using an ELISA Kit (R&D Systems, Minneapolis, MN, USA) as previously described [38,39]. 

### 2.3. Microcomputed Tomography (µCT)

Following sacrifice, left femoral bones were harvested, fixed with 10% neutral buffered formalin and then stored in phosphate buffered saline (PBS). Bone microcomputed tomography (µCT) was performed with Scanco µCT 35 (Scanco Medical, Brüttisellen, Switzerland) as previously described [14]. A 1.35-mm section of the distal part the left femur, starting 100 µm from the growth plate, was used for trabecular bone analysis. A 1.4-mm section of the mid-diaphysis was used for analysis of cortical bone. A 6-µm voxel size, 55kVp, a 0.36° rotation step (180° angular range), and a 400-ms exposure per view were used for the scans, which were performed in PBS. Scanco µCT software (HP, DECwindows Motif 1.6, Scanco Medical, Brüttisellen, Switzerland) was used for 3D reconstruction and viewing of images. After 3D reconstruction, volumes were segmented using global thresholds of 300 and 560 mg/c for trabecular and cortical bone, respectively. Trabecular bone mass (Tb.BV/TV), number (Tb.N), thickness (Tb.Th), and space (Tb.Sp) were calculated for the trabecular bone. Cortical bone mass (BV/TV), thickness (Ct.Th), and porosity (Ct.Po) were also calculated for cortical bone.

### 2.4. Molecular Assessments

After µCT, the left femurs were decalcified in 10% EDTA for 14 days and embedded in paraffin. Each section of paraffin-embedded left femur containing the metaphyseal area was deparaffinized and lysed with RNeasy FFPE kit (Qiagen, Valencia, CA, USA) to isolate total RNA. Total RNA was reverse transcribed into cDNA using the High-Capacity RNA-to-cDNA™ Kit (Applied Biosystems, Foster City, CA, USA). The cDNA was then amplified with specific mouse primers (Table 2) in the presence of the PerfeCTa^®^ Green SuperMix with Low ROX™ (QuantaBio, Beverly, MA, USA) via the QuantStudio 6 Flex (Applied Biosystems, Foster City, CA). Gene expression was normalized to that of glyceraldehyde 3-phosphate dehydrogenase (GAPDH). 

### 2.5. Mechanical Testing

The remaining right femoral bones of the mice after sacrifice were harvested and stored at −80 °C. Bone quality and bone integrity were assessed by a three-point bending system (Instron Microtester 5848, Norwood, MA, USA) [40]. A test was performed in the anterior-posterior direction using a 100 N load cell (accuracy, ± 0.025 N). A constant ramp displacement (0.05 mm/s) was used to apply a load at the midpoint of the femur until failure. Failure load and stiffness were measured. The elastic moduli were also determined using Classical Beam Theory with dimensional values of the mouse femur. Their values were obtained from morphometric µCT data using a Skyscan 1272 µCT system (Bruker, Manning Park, MA, USA). 

### 2.6. Statistical Analyses

Values are presented as mean ± SEM (*n* = 4–8). To assess the effects of treatment, a one-way analysis of variance (ANOVA) test comparing treatment groups via Prism Version 6.0f (GraphPad Software, Inc., La Jolla, CA, USA) was run. When ANOVA indicated any significant difference among the means (*p* < 0.05), Fisher’s least significant difference test, without correcting for multiple comparison, was used to determine which means were significantly different (*p* < 0.05). For analysis of mechanical testing, we calculated the combined mean of the TG + 5% TC and TG + 10% TC groups due to the limited number of samples per group.

## 3. Results

### 3.1. Although a High-Dose TC Diet Prevents TNF-Mediated Loss of Body Weight, It Does Not Suppress Elevated Proinflammatory Cytokines

**Figure 1:**
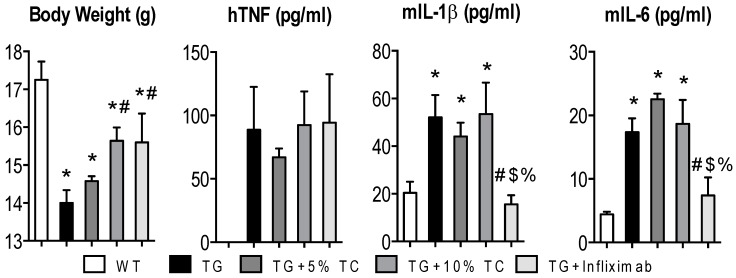
High-doses of TC recovered a TNF-mediated loss of body weight, but it did not suppress TNF-mediated elevation of proinflammatory cytokines in the blood. After 4 weeks of treatment, body weight was measured in wild-type (WT), transgenic (TG), TG with 5% TC, TG with 10% TC, and TG with infliximab mice. Collected serum after sacrifice was also used to measure protein levels of human TNF (hTNF), mouse interleukin (mIL)-1β, and mIL-6. Values are mean ± SEM (*n* = 4–8 per group). *, *p* < 0.05 vs. WT; #, *p* < 0.05 vs. TG; $, *p* < 0.05 vs. TG + 5% TC; %, *p* < 0.05 vs. TG + 10% TC.

Since we performed pair-feeding to ensure an equal amount of consumption, all mice were fed around 2 g per day. Despite this, TG mice experienced significant reduction of body weight after a four-week treatment compared to WT mice (Figure 1). However, treatment of TG mice with either 10% TC or infliximab significantly attenuated TNF-induced loss of body weight. 

We also measured protein levels of hTNF, mIL-1β, and mIL-6 in sera obtained from the WT, TG, TG treated with 5% TC, TG treated with 10% TC, and TG treated with infliximab groups. We validated that serum levels of hTNF were absent in WT and highly elevated in TG mice regardless of treatment. Furthermore, we observed that TC did not alter TNF-mediated elevation of mIL-1β and mIL-6 levels, while infliximab significantly decreased serum levels to bring them down to that of WT. These data indicate that TC prevented TNF-induced loss of body weight without modulating serum proinflammatory cytokines.

### 3.2. TC Diet Dose-Dependently Protects Trabecular Bone from Inflammation-Induced Bone Destruction

**Figure 2:**
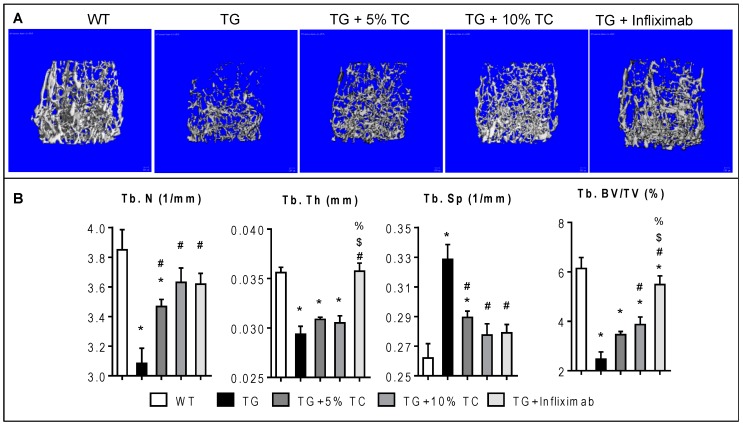
TC diet dose-dependently inhibited TNF-induced decline of trabecular bone mass. (**A**) Microcomputed tomography (µCT) scans representing metaphyseal trabecular bone were taken from the femurs of WT, TG, TG + 5% TC, TG + 10% TC, and TG + infliximab mice. (**B**) Trabecular number (Tb.N), thickness (Tb.Th), space (Tb.Sp), and bone mass (Tb.BV/TV) were quantified using µCT analysis. Values are mean ± SEM (*n* = 4–8 per group). *, *p* < 0.05 vs. WT; #, *p* < 0.05 vs. TG; $, *p* < 0.05 vs. TG + 5% TC; %, *p* < 0.05 vs. TG + 10% TC.

To examine the effects of TC on TNF-mediated alteration of microstructural morphology in trabecular bone (Figure 2), we performed µCT on femurs. In Figure 2A, TG showed a dramatic loss of metaphyseal trabecular bone mass compared to WT. However, TG fed on TC diet exhibited bone protective effects against TNF-mediated bone loss. The degree of trabecular bone loss caused by inflammation was also minimized by treatment with infliximab. 

TG mice showed significant decreases in both Tb.N and Tb.Th by 20% and 18%, respectively compared to WT mice. TNF-mediated reduction of Tb.N was significantly recovered by treatment with either TC (5% and 10%) or infliximab by 6–10% or 6%, respectively. On the other hand, only treatment with infliximab significantly restored TNF-induced narrowing of Tb.Th to the levels of WT. Additionally, TG mice showed a significantly higher Tb.Sp, at 1.25-fold over WT. Similar to treatment with infliximab, both doses of TC diet significantly inhibited a TNF-induced increase in Tb.Sp by 12% and 16%, respectively. Overall, TG mice displayed 40% of Tb.BV/TV compared to WT, while TG mice treated with either TC diet or infliximab exhibited 56–63% or 89%, respectively, compared to WT. Both imaging and quantitative data indicate that treatment with TC inhibits loss of trabecular bone mass through recovery from significant TNF-mediated reduction of the number and increase of space in the trabecular structure.

### 3.3. TC Diet Prevents a TNF-Mediated Reduction of Cortical Thickness and Increase of Cortical Porosity

**Figure 3:**
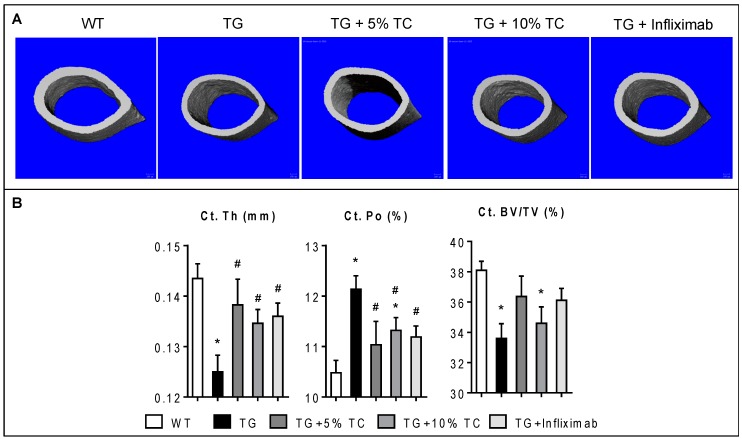
TC diet improved modification of cortical bone structure by inflammation. (**A**) Microcomputed tomography (µCT) scans representing sections of cortical bone were taken for femurs of WT, TG, TG + 5% TC, TG + 10% TC, and TG+infliximab mice. (**B**) Cortical thickness (Ct.Th), porosity (Ct.Po), and bone mass (Ct.BV/TV) were quantified using µCT analysis. Values are mean ± SEM (*n* = 4–8 per group). *, *p* < 0.05 vs. WT; #, *p* < 0.05 vs. TG.

We also assessed microstructural properties of cortical bone in the diaphyseal area of femurs (Figure 3). TG mice exhibited much thinner cortical bone compared to WT (Figure 3A). However, TG mice treated with either TC diet or infliximab showed an intermediate thickness of cortical bone, between that of WT and TG. 

Significant reduction of Ct.Th by 13% and induction of Ct.Po by 16% in TG were observed compared to WT (Figure 3B). However, treatment with either a 5% or 10% TC diet significantly prevented TNF-mediated reduction of Ct.Th, by 4% and 6%, respectively. Furthermore, the TC diet significantly decreased TNF-induced Ct.Po compared to the control diet. Similar effects were also shown in TG mice treated with infliximab. Finally, significant inflammation-mediated reduction of cortical bone mass (12%) was modestly recovered by treatment with either 5% TC or infliximab. These data indicate that TC diet modestly improved the modification of cortical bone structure caused by inflammation. 

### 3.4. TC Modulates Gene Expressions Altered by TNF during Bone Destruction

**Figure 4:**
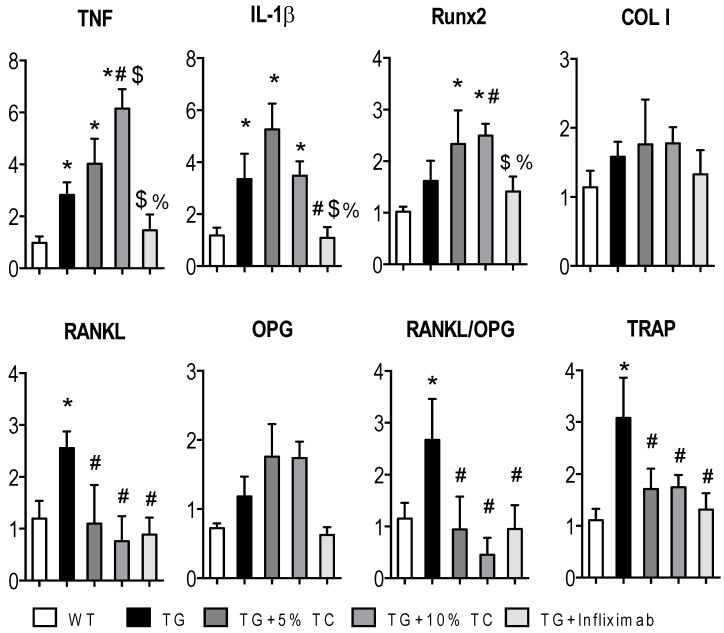
TC downregulated gene expressions of *RANKL* and *TRAP*. Total RNA was extracted from histological sections of metaphyseal area in each femur of WT, TG, TG + 5% TC, TG + 10% TC, and TG + infliximab mice. cDNA converted from total RNA was applied to measure relative transcript levels of *TNF, IL-1β, Runx2, COL I, RANKL*, and *TRAP* that were normalized to *GAPDH* using RT-PCR. Values are means (*n* = 4–8) ± SEM. *, *p* < 0.05 vs. WT; #, *p* < 0.05 vs. TG; $, *p* < 0.05 vs. TG + 5% TC; %, *p* < 0.05 vs. TG + 10% TC.

To determine the role of TC in TNF-mediated gene profiling under the condition of bone destruction, we measured the transcript levels of (1) the proinflammatory cytokines TNF and IL-1β, (2) the osteoblast markers runt-related transcription factor 2 (Runx2) and COL I, and (3) the osteoclast-associated genes tartrate-resistant acid phosphatase (TRAP), RANKL, and osteoprotegrin (OPG) (Figure 4). 

mRNA levels of *TNF* and *IL-1β* were highly expressed in TG compared to WT. While these elevated expressions were significantly further increased or maintained by treatment with TC diet, they were modestly and significantly decreased by treatment with infliximab. The transcript levels of *Runx2* were significantly and highly expressed in TG treated with TC compared to WT. Diet with 10% TC also significantly increased transcript levels of *Runx2* compared to TG group. However, its expression in TG mice treated with infliximab was brought down to levels of the WT and TG groups. Nevertheless, the expression of *COL I* was not affected by the status of TG and treatments. 

Increased transcript levels of *RANKL* in TG mice were significantly reduced by either TC or infliximab treatment without influencing that of OPG. Following a similar pattern to *RANKL*, highly expressed TNF-mediated levels of *TRAP* were significantly decreased by treatment with either TC or infliximab. The data indicate that TC diet regulates genes associated with osteoclasts and osteoblasts with an uncoupling system to have beneficial effects on bone mass. 

### 3.5. TC Diet Modestly Improves TNF-Mediated Reduction of Mechanical Strength

**Figure 5:**
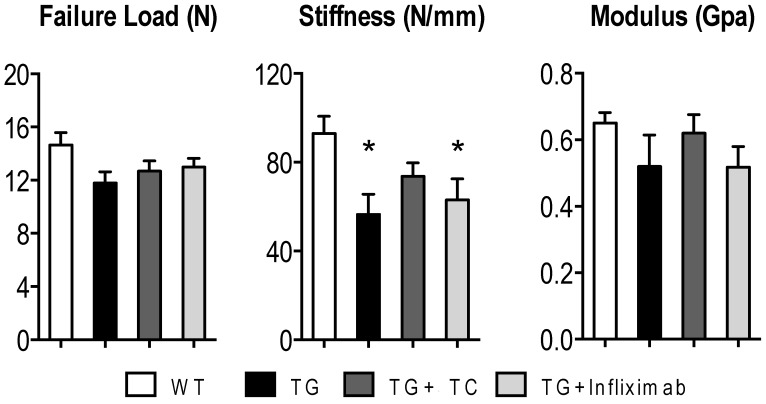
TC exhibited a modest improvement of bone stiffness devastated by TNF overexpression. Right femurs were placed on the machine with a three-point bending mechanical system to measure and determine failure load, stiffness, and modulus. The 5% and 10% TC mice were combined for these parameters to make a total of four groups: WT, TG, TG + TC, and TG + infliximab. Values are means (*n* = 4–9) ± SEM. *, *p* < 0.05 vs WT.

To determine the effects of TC on TNF-induced damage of the structural and material properties, we performed a three-point bending experiment to measure failure load, stiffness, and modulus (Figure 5). Because of the limited number of bone, we combined the two groups (TG + 5% and TG + 10%) for these analyses.

No differences were found across all groups for failure load or modulus. However, there was a significant reduction in stiffness in TG mice compared to WT by 39%. While treatment with TC diet was modestly able to prevent this reduction in stiffness, treatment with infliximab was not. This indicates that the increased bone mass mediated by treatment with TC correlates to an improvement in mechanical strength.

## 4. Discussion

The purpose of this study is to examine the effects of TC on inflammation-mediated bone loss and fragility using TNF-overexpressing mice. We demonstrated that TC protected bone mass and strength from the state of inflammation. 

Expectedly, infliximab prevented TNF-mediated bone loss and inhibited the progression of systemic inflammation. This observation has also been demonstrated in RA patients treated with TNF-targeting antibodies including infliximab by showing a great efficacy in improving bone mass as well as alleviating RA symptoms such as joint pain [2,8,9,10,11,15,16]. On the other hand, a TC diet demonstrated bone protective properties against TNF-mediated bone destruction in both trabecular and cortical bones without altering the state of inflammation. Similar results were observed in TNF overexpressing mice with treatment of denosumab (DMAB), which was recently developed in the form of an antibody against RANKL. That is, DMAB prevented bone loss without reducing TNF-mediated overproduction of inflammatory cytokines [34]. DMAB has also been tested in RA patients with osteoporosis and was shown to improve bone mass [41,42,43]. Since previous studies have found TC to lower serum protein levels of TNF as well as other proinflammatory cytokines in both human [44] and rat subjects [45], TC is likely to have a modest anti-inflammatory effect in the setting of a mild inflammatory condition. However, our data suggests that under the state of chronically high systemic overexpression of inflammatory mediators, TC regulates TNF-mediated loss of bone mass at the local rather than systemic level.

We found that TC downregulated expression of RANKL and TRAP, which are indicative of osteoclast formation. These effects of whole TC were also displayed by the individual bioactive compounds found in TC. For example, kaempferol abrogated TNF-induced nuclear factor-κB (NFκB) activation, leading to decreased formation of osteoclasts [46]. Proanthocyanidin prevented RANKL-induced osteoclast formation and activity through repression of nuclear factor of activated T-cells (cytoplasmic 1 (NFATc1)) [47]. Cyanidin also inhibits RANKL-induced osteoclast formation in preosteoclasts (RAW264.7 cells) [48]. Furthermore, we previously demonstrated that neochlorogenic acid reduced formation of osteoclasts via repression of osteoclast-specific gene expressions including TRAP and cathepsin K (CatK) stimulated by TNF in primary mouse bone marrow cells [14]. Thus, the molecular mechanism behind the effect of TC on inflammation-mediated bone loss may be working via the inhibition of osteoclast-associated gene expressions. 

Bone mass is determined by the overall balance of functions between osteoclasts and osteoblasts. TC downregulates gene expression of markers of osteoclasts while maintaining those of osteoblasts through an uncoupling scheme under the inflammatory condition. A similar pattern was previously observed for an inhibitor of CatK that was capable of improving bone mass by maintaining bone formation while inhibiting bone resorption [39]. Strontum ranelate, a drug used in the treatment of postmenopausal osteoporosis, also increased bone formation but deceased bone resorption [49,50]. Based on our data, TC uncouples osteoblasts and osteoclasts, leading to maintenance of bone mass under the inflammatory condition. 

The increased bone mass in TG mice treated with TC confers modestly improved bone strength. Interestingly, the significant improvements to bone morphology by infliximab are not associated with a similar enhancement of bone strength. Our data agreed with a population-based cohort study demonstrating that RA subjects treated with infliximab showed an increased bone mineral density, but not an improvement to bone strength compared to patients treated with methotrexate [51]. In spite of the enhanced bone mass, bisphosphonates, anti-bone resorptive drugs used in the treatment of osteoporosis, [12,52,53,54] also exhibit undesirable outcomes in bone strength and quality. For example, the continuous usage of bisphosphonates for more than 5 years was associated with increased atypical fractures at the sites of the subtrochanteric and femoral shaft in a cohort of women aged 68 years or older [55]. Furthermore, osteoporotic patients who took bisphosphates (alendronate) for several years even displayed delayed or deficient fracture healing [56]. This vulnerability in bone quality caused by bisphosphonates could be due to an accumulation of old bone matrix secondary to a decreased degree of both bone formation and resorption. Thus, TC might improve bone quality and fracture healing via maintaining bone formation by overcoming these restrictions of anti-bone resorptive drugs.

## 5. Conclusions

An intake of TC that enhanced bone mass through uncoupling of osteoclasts and osteoblasts conferred bone strength. This quality might offer the usage of a safe long-term dietary supplement for the prevention of future fragility fractures in states of high chronic inflammation. 

## Figures and Tables

**Table 1:** Diet composition. TC: tart cherry.

Ingredient		^1^ 0%	5%	10%
		g/kg Diet		
TC Carbohydrates Proteins Fat Fiber ^2^ Mineral mix ^3^ Vitamin mix Choline bitartrate L-cystine ^4^ TBHQ, antioxidants	Cornstarch Maltodextrin Sucrose Casein Soybean oil Cellulose	0 397.5 132 100.00 200 70.00 50.00 35 10 2.5 3 0.014	50 391.3 132 66.36 194.8 69.59 45.49 35 10 2.5 3 0.014	100 385.1 132 32.72 189.5 69.18 40.97 35 10 2.5 3 0.014
Calculated nutrients				
Kcal/g		4	4	4
Macronutrients, % by weight				
Protein Carbohydrate Fat Sugar (sucrose + TC)		18 60 7 13	18 60 7 13	18 60 7 13

^1^ AIN-93G diet (Harlan, Indianapolis, IN, USA). ^2^ AIN-93G-MX: 27.26 g of mineral mix and 7.74 g of sugar. ^3^ AIN-93-VX: 0.25 g of vitamin mix and 9.75 g of sugar. ^4^ TBHQ: Tertiary butylhydroquinone.

**Table 2:** Mouse primer sequences. TNF: tumor necrosis factor; IL: interleukin; COL I: type I collagen; RANKL: receptor activator of nuclear factor kappa-B ligand; OPG: osteoprotegerin; TRAP: tartrate-resistant acid phosphatase; GAPDH: glyceraldehyde 3-phosphate dehydrogenase

Target Name	Forward Primer (5′-3′)	Reverse Primer (5′-3′)
TNF IL-1β Runx2 COL I RANKL OPG TRAP GAPDH	CCCCAAAGGGATGAGAAGTT ATGAAGGGCTGCTTCCAAA CCACCACTCACTACCACACG ACTGGTACATCAGCCCGAAC CAGAAGGAACTGCAACACATTG GTGTGGAATAGATGTCACCCT GATGACTTTGCCAGTCAGCA GGTCGGTGTGAACGGATTT	GGTCTGGGCCATAGAACTGA GGACAGCCCAGGTCAAAG CACTCTGGCTTTGGGAAGAG AATCCATCGGTCATGCTCTC CTCCTGAGAAGCGCTGTG AAGAAGGCCTCTTCACACAG AACTGCTTTTTGAGCCAGGA GACCAGGCGCCCAATAC
